# P-2277. Impacts of Ongoing Quinolone Prophylaxis Following Quinolone-Resistant Bacteremia in Neutropenic Patients

**DOI:** 10.1093/ofid/ofae631.2430

**Published:** 2025-01-29

**Authors:** Sarah Acevedo, Stacy Park

**Affiliations:** University of Virginia Health, Charlottesville, Virginia; University of Virginia Medical Center, Charlottesville, VA

## Abstract

**Background:**

Guidelines that suggest fluoroquinolone (FQ) prophylaxis for intermediate to high-risk hematologic malignancy patients are based primarily on evidence of reduction in bloodstream infections (BSIs) in historic studies, despite no demonstrated reduction in other outcomes such as mortality, septic shock, or ICU admissions. In the era of widespread FQ use for prophylaxis and increasing antimicrobial resistance, ongoing re-evaluation of the benefit is needed. Patients with BSI due to FQ-resistant gram-negative organisms may have particularly reduced benefit from ongoing FQ prophylaxis, however data on this topic is limited. We evaluated characteristics of neutropenic patients with BSI in an attempt to better inform the risk/benefit balance in this population.

Figure 1.

**Figure:**
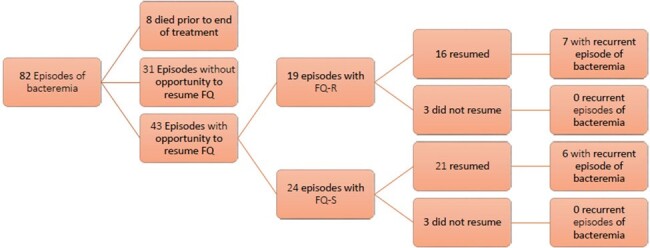

Numerical breakdown of bacteremia episodes

**Methods:**

We extracted episodes of gram-negative BSI during a neutropenic episode lasting at least 7 days in adult inpatients with hematologic malignancy and/or stem cell transplant (study period 11/1/2020-11/1/2023). For each BSI episode we collected data on treatment (agent, duration, line removal), FQ susceptibility, underlying disease, neutropenia, and FQ exposure within 90 days. For episodes with an opportunity to resume prophylaxis following treatment of bacteremia (based on persistent neutropenia or recurrent neutropenia with 90 days), we determined rates of recurrent BSI within 90 days, stratified by FQ resistance and decision to resume FQ prophylaxis.

**Results:**

There were 82 BSI episodes (69 first episodes, 13 recurrent) among 62 patients. FQ resistance was common in first episodes (34/69, 49%) and median prior FQ exposure within 90 days was 16 days. 43 episodes had an opportunity to resume FQ prophylaxis and 19/43 (44%) had FQ resistance. FQ prophylaxis was resumed in 16/19 (84%) with FQ resistance and 21/24 (88%) without, with rates of recurrent bacteremia within 90 days of 44% and 29% respectively. There were no episodes of recurrent bacteremia when FQ prophylaxis was not resumed (0/6).

**Table 1.**
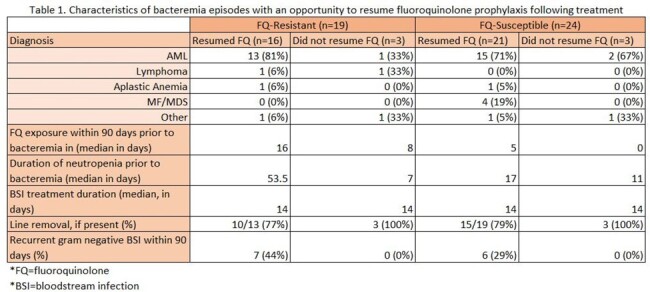
Characteristics of bacteremia episodes with an opportunity to resume fluoroquinolone prophylaxis following treatment

**Conclusion:**

Among bacteremia episodes with eligibility for FQ prophylaxis following treatment, recurrent bacteremia was common, particularly when FQ resistance was present. FQ prophylaxis may have little or no benefit in this setting, however larger studies and controlled data are needed.

**Disclosures:**

All Authors: No reported disclosures

